# The accuracy of artificial intelligence used for non-melanoma skin cancer diagnoses: a meta-analysis

**DOI:** 10.1186/s12911-023-02229-w

**Published:** 2023-07-28

**Authors:** Kuang Ming Kuo, Paul C. Talley, Chao-Sheng Chang

**Affiliations:** 1 Department of Business Management, National United University, No.1, Miaoli 360301 Lienda, Taiwan, Republic of China; 2 Department of Applied English, I-Shou University, No. 1, Sec. 1, Syuecheng Rd., Dashu District, 84001 Kaohsiung City, Taiwan, Republic of China; 3 Department of Occupational Therapy, I-Shou University, No. 1, Yida Rd., Yanchao District, 82445 Kaohsiung City, Taiwan, Republic of China; 4 Department of Emergency Medicine, E-Da Hospital, I-Shou University, Kaohsiung, Taiwan, Republic of China

**Keywords:** Artificial intelligence, Cross validation, Diagnostic test accuracy, Ensemble models, Meta-analysis, Non-melanoma skin cancers, Pre-trained models

## Abstract

**Background:**

With rising incidence of skin cancer and relatively increased mortality rates, an improved diagnosis of such a potentially fatal disease is of vital importance. Although frequently curable, it nevertheless places a considerable burden upon healthcare systems. Among the various types of skin cancers, non-melanoma skin cancer is most prevalent. Despite such prevalence and its associated cost, scant proof concerning the diagnostic accuracy via Artificial Intelligence (AI) for non-melanoma skin cancer exists. This study meta-analyzes the diagnostic test accuracy of AI used to diagnose non-melanoma forms of skin cancer, and it identifies potential covariates that account for heterogeneity between extant studies.

**Methods:**

Various electronic databases (*Scopus, PubMed, ScienceDirect, SpringerLink*, and *Dimensions*) were examined to discern eligible studies beginning from March 2022. Those AI studies predictive of non-melanoma skin cancer were included. Summary estimates of sensitivity, specificity, and area under receiver operating characteristic curves were used to evaluate diagnostic accuracy. The revised Quality Assessment of Diagnostic Studies served to assess any risk of bias.

**Results:**

A literature search produced 39 eligible articles for meta-analysis. The summary sensitivity, specificity, and area under receiver operating characteristic curve of AI for diagnosing non-melanoma skin cancer was 0.78, 0.98, & 0.97, respectively. Skin cancer typology, data sources, cross validation, ensemble models, types of techniques, pre-trained models, and image augmentation became significant covariates accounting for heterogeneity in terms of both sensitivity and/or specificity.

**Conclusions:**

Meta-analysis results revealed that AI is predictive of non-melanoma with an acceptable performance, but sensitivity may become improved. Further, ensemble models and pre-trained models are employable to improve true positive rating.

**Supplementary Information:**

The online version contains supplementary material available at 10.1186/s12911-023-02229-w.

## Background

Skin cancer accounts for 32.5% of all diagnosed malignancies, and it has a prevalence of 7.96 million cases occurring globally each year among the general population [1]. With respect to etiology, previous studies have demonstrated a deleterious association with chronic exposure to sunlight because the ultraviolet component induces deoxyribonucleic acid damage which later triggers malignant mutations to occur. Other possible contributors to skin cancer incidence may also include viral infection, drug usage and exposure to chemicals [2].

Pathologically, skin cancer is categorized into either melanoma or non-melanoma. Albeit relatively rare, three hundred thousand annual cases of melanoma are determined as highly malignant, with a reported mortality rate of 1.6 per 100,000 worldwide [1]. By contrast, non-melanoma cases, which comprise a number of pathologically-distinct entities such as basal cell carcinoma and intra-epithelial carcinoma (i.e., actinic keratosis and Bowen’s disease) [3], are less malignant considering Mohs micrographic surgery and a 5-year cure rate of 98.9% [4]. Sixty-five thousand victims die on average, per annum, worldwide due to non-melanoma incidence when combined with a delayed diagnosis factor [1]. Furthermore, non-melanoma skin cancers such as basal cell carcinoma show a trend of increasing cases [5] and are easily misdiagnosed [6]. The abovementioned evidence clearly shows the diagnosis of non-melanoma skin cancer is of similar importance to melanoma forms of skin cancer.

Currently, clinical examination and dermoscopic evaluation are major techniques for screening skin cancers [7]. These screening techniques are estimated to achieve 75–84% of diagnosis accuracy, indicating human error may remain accountable via these approaches [8, 9]. When taking into account the high prevalence and life-threatening risk of this disease, it is important to make a timely diagnosis for appropriate treatment to follow.

Artificial intelligence (AI) techniques are being employed to provide diagnostic assistance to dermatologists since most diagnoses rely principally on visual patterning recognition [10], a particular strength of such a technology. Machine learning is a sub-field of AI which refers to an effort to automate intellectual tasks normally performed by humans; and, deep learning is in turn a subset within machine learning [11]. A veritable plethora of attempts to utilize machine learning techniques aimed at supporting the accurate diagnosis of melanoma and non-melanoma types of skin cancer have already taken place [9, 12–34]. As such, a systematic reporting is deemed necessary for reliable interpretation and aggregation of these results. However, the comparison of pre-existing skin lesion classification evidence is difficult because differences may exist in the data types used or in the statistical quantities presented [35].

Until present time, synthetic evidence regarding the performance of AI techniques applied for the diagnosis of non-melanoma skin cancer remains insufficient [7, 10]. Without reliable evidence, the application of AI in the diagnosis of non-melanoma skin cancer is frequently obstructed. Furthermore, what important factors/strategies that may influence the performance of AI in the diagnosis of non-melanoma skin cancer are at times unclear.

In viewing the unfulfilled areas of knowledge, the purposes of this meta-analysis are therefore: 1) to meta-analyze the accuracy of diagnosis for non-melanoma skin cancer via machine learning and deep learning; and, 2) to examine potential covariates that can account for the heterogeneity found among these studies. The main contributions of this study are:

- Summary of the performance of AI for diagnosing non-melanoma skin cancer with quantitative evidence so that AI’s utility assessment can be made with greater efficacy and objectivity.
- Identification of potential covariates as they relate to AI performance since it may improve through an adoption of those strategies indicated by these identified covariates whenever building AI models.
- Accumulation of knowledge of diagnostic test accuracy for AI in non-melanoma skin cancer takes place so that earlier and more accurate diagnosis of non-melanoma skin cancer is practical.

The remainder of this paper is structured as follows. Related work section introduces prior reviews on the topic of diagnostic test accuracy, focusing on how these reviews were planned and evaluated. Material and methods section presents the research method adopted in this study. Results section describes the analytical findings based on collected data, Discussion section interprets and describes the significance of the findings, and Conclusions section summarizes the findings of the current study.

### Related work

Up until the most recent examples, a number of studies have started to review existing evidence related to AI techniques for skin-lesion classification [7, 10, 23, 35–37]. Several themes may be observed from Table 1. First, much evidence is qualitative in nature [10, 35–37], except for the study of Sharma et al. [7] and Rajpara et al. [23]. Without quantitative evidence, the performance of AI-based predictive models are not easily or objectively assessed. Second, few reviews [7, 10] have focused solely on non-melanoma forms of skin cancer, with such efforts being devoted to the review of evidence concerning melanoma [16, 23] or both [35, 37]. By focusing exclusively on non-melanoma skin cancer, a better understanding may yet be achieved. Third, most reviews include studies that have adopted machine learning and deep learning, with the exception of Brinker et al. [35]. Despite deep learning being widely considered as having better performance than machine learning, studies that adopted machine learning should also be included in order to have a more holistic understanding of AI performance in the diagnosis of melanoma and non-melanoma skin cancers. Finally, review components/metrics for assessing the performance of AI techniques are quite diversified. Classification methods, data source, and diagnostic accuracy are primary components of these reviews. Further, reviews that followed the Preferred Reporting Items for a Systematic Review and Meta-analysis statement (PRISMA) for Diagnostic Test Accuracy (DTA) commonly reported pooled diagnostic odds ratio, pooled positive/negative likelihood ratio, pooled sensitivity, and pooled specificity, while other reviews usually reported separate accuracy, area under receiver characteristic curve, F1-score, precision, sensitivity, or specificity by individual study. This study therefore follows PRISMA-DTA for reporting summary metrics of included studies for global assessment of AI performance for the diagnosis of non-melanoma skin cancer.

**Table 1:** Prior reviews on skin cancer diagnosis based on artificial intelligence

Sources	Type of skin cancer	Method	AI techniques	Studies	Review component(s)	Metrics	Main findings
[23]	Melanoma	Quantitative	ML and DL	30	Comparison between dermoscopy and AI	• DOR • ± LR • Sensitivity • Specificity	• Pooled DOR of AI: 57.80 • Pooled + LR of AI: 6.36 • Pooled -LR of AI: 0.13 • Pooled sensitivity of AI = 0.91 • Pooled specificity of AI = 0.79
[35]	Both	Qualitative	DL	13	Methods of classification	• Accuracy • AUROC • Sensitivity • Specificity	• Accuracy: 0.50–0.93 • AUROC: 0.82–0.98 • Sensitivity: 0.58–0.95 • Specificity: 0.74–0.85 • CNN can be used to extract features or classify lesions • The comparison of different AI procedures is difficult
[10]	Non-melanoma	Qualitative	ML and DL	39	• Skin lesion database • Feature extraction • Methods of classification • Diagnostic accuracy	• Accuracy • AUROC	• Accuracy: 0.72–1 • AUROC: 0.83–1
[7]	Non-melanoma	Quantitative	ML and DL	57	• Skin cancer type • Algorithm type • Diagnostic standard • Data set source and size	• Sensitivity • Specificity	• Pooled sensitivity = 0.89 • Pooled specificity = 0.81 • ML algorithms for diagnosing skin cancer is promising
[36]	Both	Qualitative	ML and DL	102	• Contributions • Methods • Achieved results	• Accuracy • Precision • Sensitivity • Specificity	• Accuracy: 0.64–1 • Sensitivity: 0.35–1 • Specificity: 0.60–1 • Precision: 0.62–0.98 • The need for creation of large public image datasets • Including clinical data may help improve classification accuracy
[37]	Melanoma	Qualitative	ML and DL	53	• Data characteristics • Diagnostic techniques • Evaluation metrics	• Accuracy • AUROC • F1-score • Sensitivity • Specificity	• Accuracy: 0.67–0.99 • AUROC: 0.82–0.92 • F1-score: 0.83 • Sensitivity: 0.77–0.96 • Specificity: 0.70–0.96

*AI* Artificial intelligence, *AUROC* Area under receiver operating characteristic curve, *CNN* Convolutional neural network, *DL* Deep learning, *DOR* Diagnostic odds ratio, *ML* Machine learning, ± *LR* Positive/negative likelihood ratio

## Methods

This study was conducted according to the PRISMA statement [38] (see Additional file 1: Appendix A for diagnostic test accuracy checklist and Additional file 2: Appendix B for diagnostic test accuracy abstracts checklist). The Institutional Review Board of E-Da Hospital (EMRP-108–128) approved the study-wide protocol.

### Search strategy and selection process

A literature search, carried out 31^st^ March, 2022, of *Scopus, PubMed, ScienceDirect, SpringerLink,* and Dimensions, by means of keyword combinations of the terms "basal cell carcinoma", "intra-epithelial carcinoma", "Bowen’s disease", "actinic keratosis", "skin lesion", "non-melanoma skin cancer", "artificial intelligence", "machine learning", and "deep learning".

**Fig. 1:**
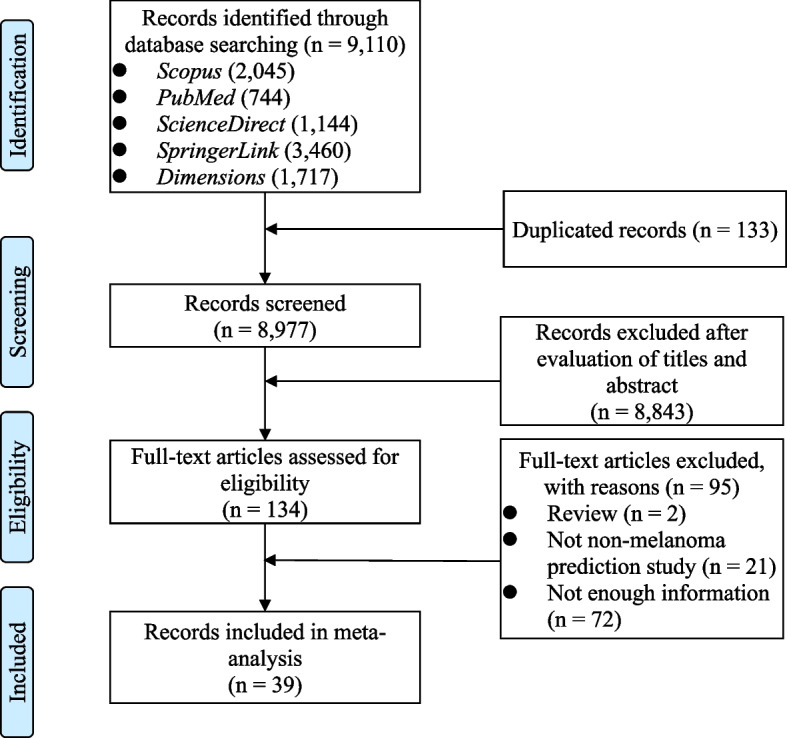
Article selection process

Inclusion criteria was determined by: 1) studies investigating the accuracy of non-melanoma skin cancer; 2) studies written in English; and, 3) studies adopting machine-learning or deep-learning techniques. Studies were dis-qualified for inclusion, if: 1) they only investigated the incidence of melanoma skin cancer; 2) studies were irrelevant to our research purpose; and, 3) full texts were unavailable for purposes of examination. We located 134 potentially eligible articles, of which 95 were excluded with reason (see Fig. 1), and the remaining 39 articles being included in the eventual quantitative meta-analysis that was made.

### Data extraction

From each study, we extracted the following information: Authorship, publication year, sample size, types of non-melanoma skin cancer described, whether data sources were publicly available, whether cross-validation procedures were undertaken, whether ensemble models were employed, and what type of artificial intelligence technique was employed (i.e., deep learning or machine learning). Only studies that adopted a neural network algorithm with more than one hidden layer were categorized as being part of the deep learning group, with others categorized as being part of the machine learning group for purposes of our study. For models based on deep learning, further recorded information including whether pre-trained models were utilized and whether image augmentation was implemented. Further, we extracted the original numbers of *true/false* positives and *true/false* negatives from each study to derive outcome measures, including summary sensitivity, specificity, and area under receiver operating characteristic curve, for purposes of diagnostic accuracy. Finally, if an article had classified more than one non-melanoma skin cancer simultaneously, we considered each of the non-melanoma skin cancers as a different study, with relevant data extracted based upon the above-listed procedures.

### Methodological analysis

Regarding the quality of each of the included studies, we evaluated the risk of bias and applicability in accordance with the revised Quality Assessment of Diagnostic Studies (QUADAS-2) including four domains: sample selection, index test, reference standard, flow, and timing [30].

### Statistical analysis

Following the suggestion of prior evidence [39], sensitivity and specificity were pooled with a bivariate model. Area under receiver operating characteristic curve, diagnostic odds ratio, positive likelihood ratio, and negative likelihood ratio were also estimated in this study. Forest plots were produced to depict variability amongst the studies up for consideration. Besides, summary receiver operating characteristic curves with 95% confidence intervals (CI) and 95% prediction intervals (PI) were adopted to assess the existence of a threshold effect among the included studies [40]. The R statistics [41] with lme4 [42] and mada [43] packages were used for diagnostic accuracy test meta-analysis.

Several meta-regressions with plausible covariates, including types of non-melanoma skin cancer (i.e., basal cell carcinoma and intra-epithelial carcinoma), whether data sources were publicly available (public or proprietary), whether cross-validation procedures were undertaken, whether ensemble models were adopted, types of AI technique employed (machine learning or deep learning), whether pre-trained deep learning models (e.g., *DenseNet, ResNet*, or *AlexNet*) were used (*Yes* or *No*), and whether image augmentation procedures were used by deep learning models (*Yes* or *No*) were undertaken to check for possible heterogeneity among studies. The significance level is set to 0.05 for present study.

## Results

### General study characteristics

Among the 39 included articles, 13 articles [6, 19, 20, 24, 26, 29, 32, 44–49] reported the identification of only one type of non-melanoma skin cancer, while other 26 articles simultaneously reported the identification of more than one non-melanoma skin cancers. Totally, 67 studies were included in our meta-analysis of diagnostic test accuracy.

Half of the included studies aimed to identify images of basal cell carcinoma (56.72%) and then intra-epithelial carcinoma (43.28%). About 76% of datasets were publicly available. Cross-validation procedures were adopted by near 22% of included studies, while approaches of ensemble models were employed by about 16%. Deep learning techniques (76%) were adopted more frequently than machine learning techniques (19%). Among 51 studies adopting deep learning, about 73% of these utilized pre-trained models for prediction, while nearly 65% of studies employed image augmentation techniques when building predictive models. Detailed characteristics of the inclusive studies is given in Tables 2 and 3, respectively.

**Table 2:** Characteristics of included studies

Characteristics	Values	Frequency	%
Study type (*n* = 67)	Basal cell carcinoma	38	56.72
	Intra-epithelial carcinoma	29	43.28
Data sources (*n* = 67)	Proprietary	16	23.88
	Public	51	76.12
Cross validation (*n* = 67)	No	52	77.61
	Yes	15	22.39
Ensemble models (*n* = 67)	No	56	83.58
	Yes	11	16.42
Types of technique (*n* = 67)	Deep learning	51	76.12
	Deep learning + Machine learning	3	4.48
	Machine learning	13	19.40
Pre-trained models (*n* = 51)	No	14	27.45
	Yes	37	72.55
Image augmentation (*n* = 51)	No	18	35.29
	Yes	33	64.71

**Table 3:** Attributes of included articles

Studies	Study type	Data sources	Cross validation	Ensemble models	Type of techniques	Pre-trained models	Image augmentation	Number of lesions	Reference standards	Techniques
[44]	BCC	Public	Y	N	DL	Y	Y	395	Pathological examination	Dermoscopic images
[50]	BCC + IEC	Public	N	N	DL	Y	N	200	Pathological examination	Dermoscopic images
[51]	BCC + IEC	Public	Y	N	DL	N	Y	4766	Pathological examination	Dermoscopic images
[52]	BCC + IEC	Public	N	N	DL	Y	Y	1981	Pathological examination	Dermoscopic images
[53]	BCC + IEC	Public	N	N	DL	Y	Y	2003	Pathological examination	Dermoscopic images
[13]	BCC + IEC	Proprietary	Y	N	ML	N	N	960	Clinical and pathological examinations	Dermoscopic images
[54]	BCC + IEC	Public	N	N	DL	N	Y	5066	Pathological examination	Dermoscopic images
[55]	BCC + IEC	Public	N	N	DL	Y	Y	1502	Pathological examination	Dermoscopic images
[56]	BCC + IEC	Public	N	N	DL	Y	N	589	Pathological examination	Dermoscopic images
[45]	IEC	Public	N	N	DL	N	Y	1200	Pathological examination	Dermoscopic images
[46]	BCC	Proprietary	N	N	DL	Y	Y	180	Unclear	Dermoscopic images
[57]	BCC + IEC	Public	N	N	DL	Y	N	1536	Unclear	Unclear
[47]	BCC	Proprietary	N	N	DL	Y	N	825	Pathological examination	Digital camera
[58]	BCC + IEC	Public	N	N	DL	Y	Y	604	Pathological examination	Dermoscopic images
[59]	BCC + IEC	Proprietary	Y	N	ML	N	N	78	Histopathology	Optical coherence tomography
[20]	BCC	Proprietary	N	N	DL	N	N	450	Histopathology	Dermoscopic images
[19]	BCC	Proprietary	Y	Y	ML	N	N	664	Expert opinions	Dermoscopic images
[60]	BCC + IEC	Public	N	Y	DL	Y	Y	1006	Pathological examination	Dermoscopic images
[61]	BCC + IEC	Public	Y	Y	DL + ML	Y	N	1002	Pathological examination	Dermoscopic images
[48]	BCC	Proprietary	Y	N	ML	N	N	520	Histology	Optical coherence tomography
[62]	BCC + IEC	Public	Y	N	DL	Y	Y	2003	Pathological examination	Dermoscopic images
[63]	BCC + IEC	Public	N	N	ML	N	N	480	Pathological examination	Dermoscopic images
[64]	BCC + IEC	Public	N	Y	DL	Y	Y	25,331	Pathological examination	Dermoscopic images
[65]	BCC + IEC	Public	N	N	DL	Y	Y	552	Pathological examination	Dermoscopic images
[66]	BCC + IEC	Public	N	N	DL	Y	N	1002	Pathological examination	Dermoscopic images
[67]	BCC + IEC	Public	N	N	DL	Y	Y	2003	Pathological examination	Dermoscopic images
[68]	BCC + IEC	Public	Y	Y	DL	Y	Y	1642	Pathological examination	Dermoscopic images
[69]	BCC + IEC	Public	N	N	DL	N	N	938	Pathological examination	Dermoscopic images
[49]	BCC	Public	N	Y	DL + ML	N	Y	4817	Pathological examination	Dermoscopic images
[70]	BCC + IEC	Public	N	N	DL	N	Y	802	Pathological examination	Dermoscopic images
[24]	BCC	Proprietary	Y	Y	ML	N	N	964	Unclear	Dermoscopic images
[26]	IEC	Proprietary	N	N	ML	N	N	373	Expert opinions	Photographic images
[27]	BCC + IEC	Public	N	N	ML	N	N	3004	Pathological examination	Dermoscopic images
[71]	BCC + IEC	Public	N	N	DL	Y	Y	2019	Pathological examination	Dermoscopic images
[29]	BCC	Public	N	N	ML	N	N	56	Pathological examination	Dermoscopic images
[72]	BCC + IEC	Public	N	N	DL	N	Y	2153	Pathological examination	Dermoscopic images
[31]	BCC + IEC	Proprietary	N	N	DL	Y	N	2909	Histopathology	Dermoscopic images
[32]	BCC	Proprietary	Y	N	DL	Y	N	507	Dermatologists	Dermoscopic images
[6]	BCC	Proprietary	N	N	DL	Y	N	1150	Dermatologists	Dermoscopic images

*BCC* Basal cell carcinoma, *IEC* Intra-epithelial carcinoma, *DL* Deep learning, *ML* Machine learning

### Quality assessment

**Fig. 2:**
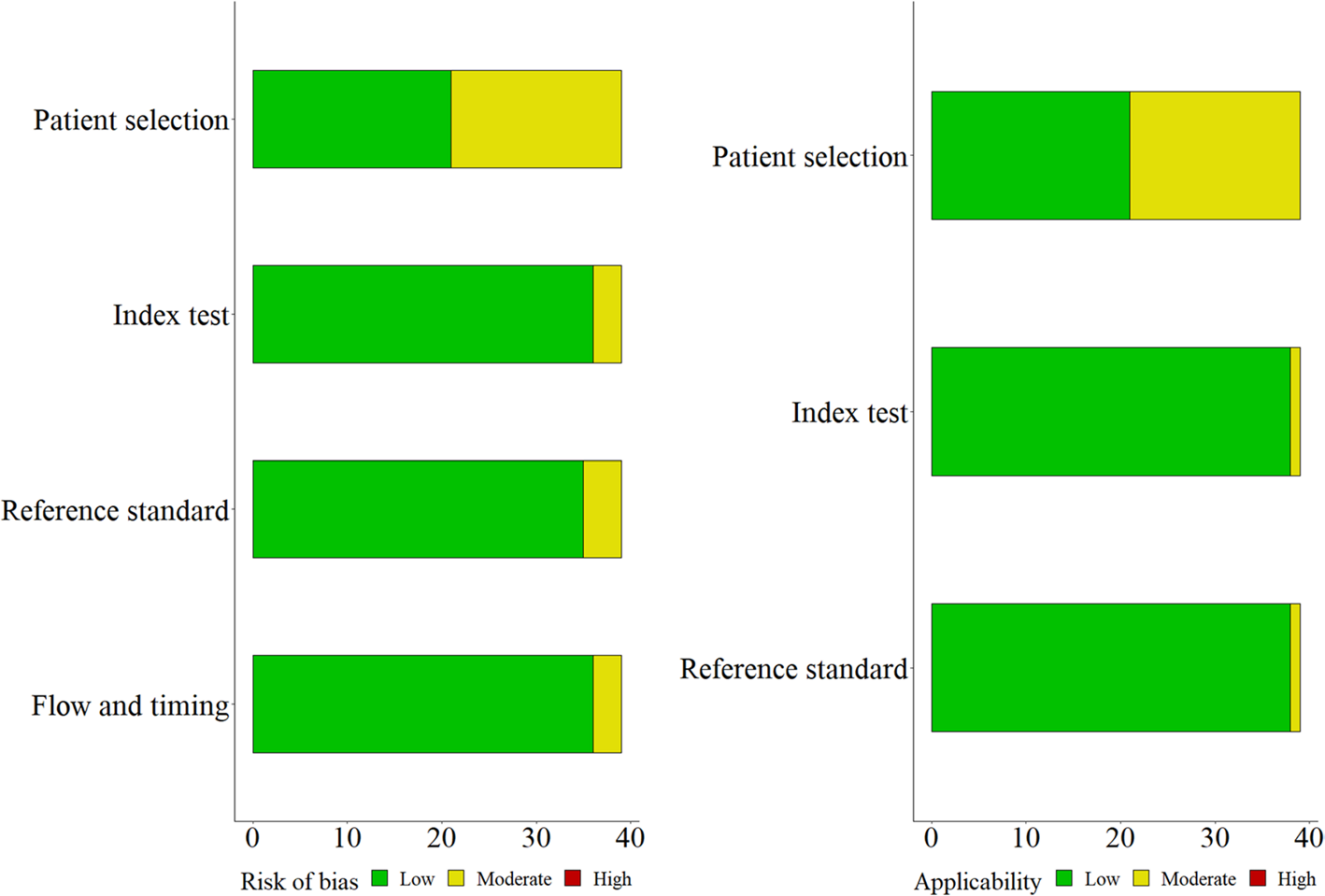
Methodological assessment by QUADAS-2

According to the QUADAS-2 tool [30], 18 out of the 39 included articles were assessed to have a moderate risk of bias, and 21 articles were considered as possessing a low risk of bias regarding patient selection. Three, four, and three articles were assessed as having a moderate risk factor related to index test, reference standard, and flow and timing, respectively (see Fig. 2). Furthermore, 18 out of 39 included articles purporting a moderate risk are regarding the applicability of patient selection processes. Further, 38 articles were categorized as having a low applicability risk about the given index test and reference standard applied.

### Diagnostic accuracy of non-melanoma skin cancer predictive models based on machine learning

**Fig. 3:**
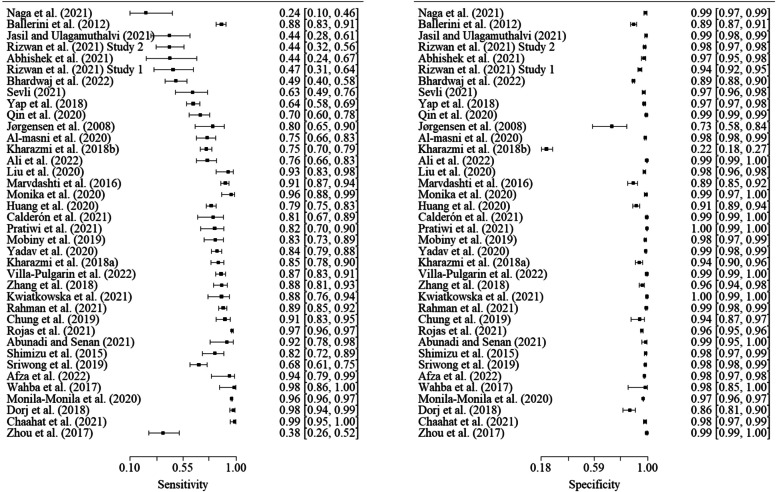
Sensitivity and specificity of basal cell carcinoma studies (*n* = 38)

**Fig. 4:**
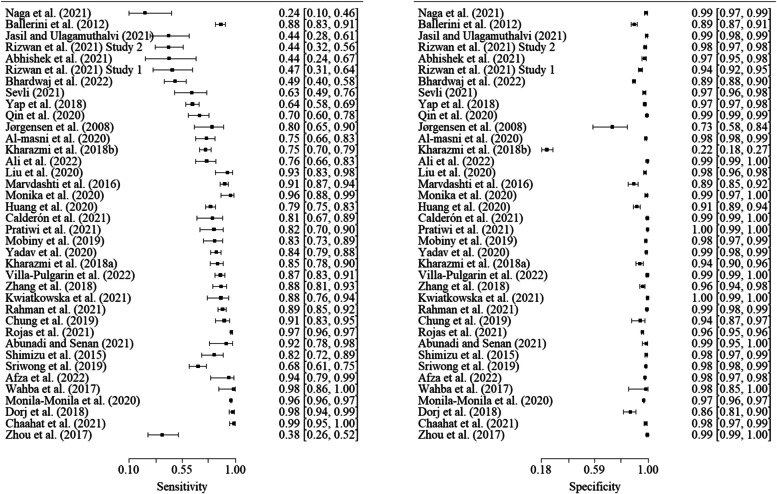
Sensitivity and specificity of intra-epithelial carcinoma studies (*n* = 29) Plausible covariates to explain between-study heterogeneity

As shown in Table 4, the overall summary area under receiver operating characteristic curve for machine learning to predict non-melanoma skin cancers is about 0.97. Moreover, summary sensitivity, specificity, diagnostic odds ratio, positive likelihood ratio, and negative likelihood ratio were 0.78, 0.98, 224.31, 49.75, and 0.22 respectively. Since our study focused on non-melanoma skin cancers, Figs. 3 and 4 illustrate the sensitivity and specificity of each of the included studies for basal cell carcinoma and intra-epithelial carcinoma, respectively.

**Table 4:** Performance of predicting non-melanoma by artificial intelligence

Metrics	Performance (95% CI)
Area under receiver operating characteristic curve	0.97
Sensitivity	0.78 (0.71, 0.84)
Specificity	0.98 (0.98, 0.99)
Diagnostic odds ratio	224.31 (123.15, 325.46)
Positive likelihood ratio	49.75 (33.94, 65.56)
Negative likelihood ratio	0.22 (0.15, 0.29)

*CI* Confidence interval

**Fig. 5:**
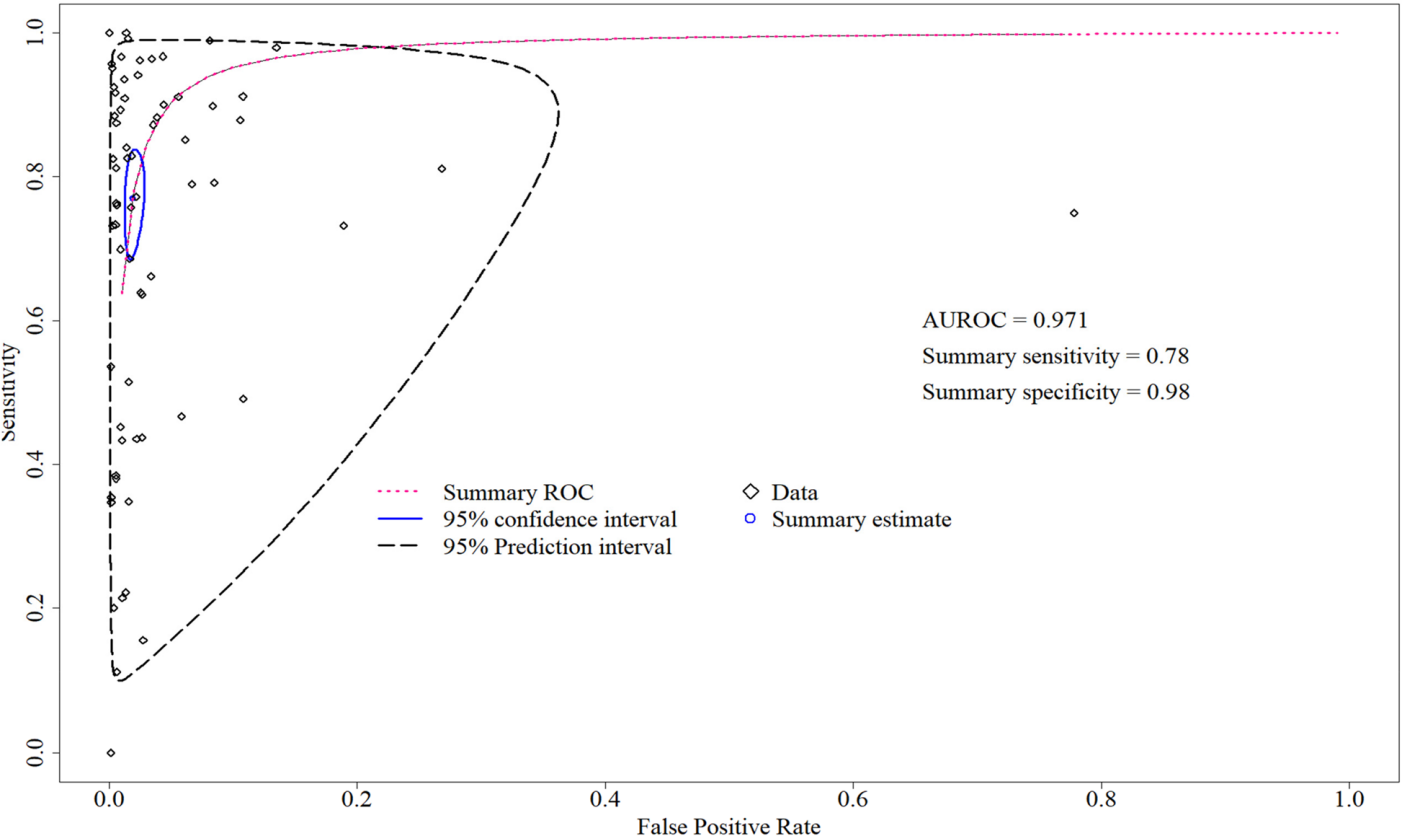
Summary receiver operating characteristic curve for overall studies

Pooling sensitivity and specificity based on univariate meta-analysis of diagnostic test accuracy can sometimes be misleading [40]. The bivariate meta-analysis of summary sensitivity, specificity, and their respective confidence intervals is depicted in Table 5. Figure 5 illustrates the summary ROC curve with 95% CI and 95% PI for included studies. We conducted χ^2^ tests to evaluate the heterogeneity of sensitivity and specificity. The test for equality of sensitivity and of specificity were both significant, χ^2^ (66) = 2702.33, *p* < 0.001 and χ^2^ (66) = 12,373.02, *p* < 0.001, indicating significant between-study heterogeneity existed for both sensitivities and specificities.

**Table 5:** Summary estimates for sensitivity and specificity

Covariates	Values	Metrics	Summary estimates	95% CI	*p* value
Overall (*n* = 67)		Sensitivity	0.78	[0.71, 0.84]	
		Specificity	0.98	[0.98, 0.99]	
Types of non-melanoma skin cancer	Basal cell carcinoma (*n* = 38)	Sensitivity	0.83	[0.77, 0.88]	[Reference]
		Specificity	0.98	[0.96, 0.98]	[Reference]
	Intra-epithelial carcinoma (*n* = 29)	Sensitivity	0.70	[0.53, 0.82]	0.046*
		Specificity	0.99	[0.98, 0.99]	0.006**
Data sources	Public (*n* = 51)	Sensitivity	0.79	[0.69, 0.86]	[Reference]
		Specificity	0.99	[0.98, 0.99]	[Reference]
	Proprietary (*n* = 16)	Sensitivity	0.78	[0.69, 0.86]	0.686
		Specificity	0.93	[0.88, 0.97]	0.000***
Cross validation	Yes (*n* = 15)	Sensitivity	0.79	[0.64, 0.89]	[Reference]
		Specificity	0.97	[0.92, 0.98]	[Reference]
	No (*n* = 52)	Sensitivity	0.78	[0.69, 0.85]	0.380
		Specificity	0.99	[0.98, 0.99]	0.008**
	Yes (*n* = 11)	Sensitivity	0.91	[0.84, 0.95]	[Reference]
		Specificity	0.98	[0.94, 0.99]	[Reference]
Ensemble models	No (*n* = 56)	Sensitivity	0.75	[0.66, 0.82]	0.014**
		Specificity	0.98	[0.98, 0.99]	0.510
Types of technique	Deep learning (*n* = 51)	Sensitivity	0.75	[0.66, 0.83]	[Reference]
		Specificity	0.99	[0.98, 0.99]	[Reference]
	Machine learning (*n* = 13)	Sensitivity	0.83	[0.69, 0.91]	0.344
		Specificity	0.95	[0.88, 0.98]	0.001**
Pre-trained models	Yes (*n* = 36)	Sensitivity	0.81	[0.73, 0.87]	[Reference]
		Specificity	0.99	[0.98, 0.99]	[Reference]
	No (*n* = 15)	Sensitivity	0.58	[0.33, 0.79]	0.048*
		Specificity	0.98	[0.97, 0.99]	0.263
Image augmentation	Yes (*n* = 34)	Sensitivity	0.73	[0.62, 0.82]	[Reference]
		Specificity	0.99	[0.99, 0.99]	[Reference]
	No (*n* = 17)	Sensitivity	0.80	[0.61, 0.91]	0.413
		Specificity	0.98	[0.96, 0.99]	0.031*

^*^denotes *p* < 0.05, ** *p* < 0.01, *** *p* < 0.001, and *CI* Confidence interval

**Fig. 6:**
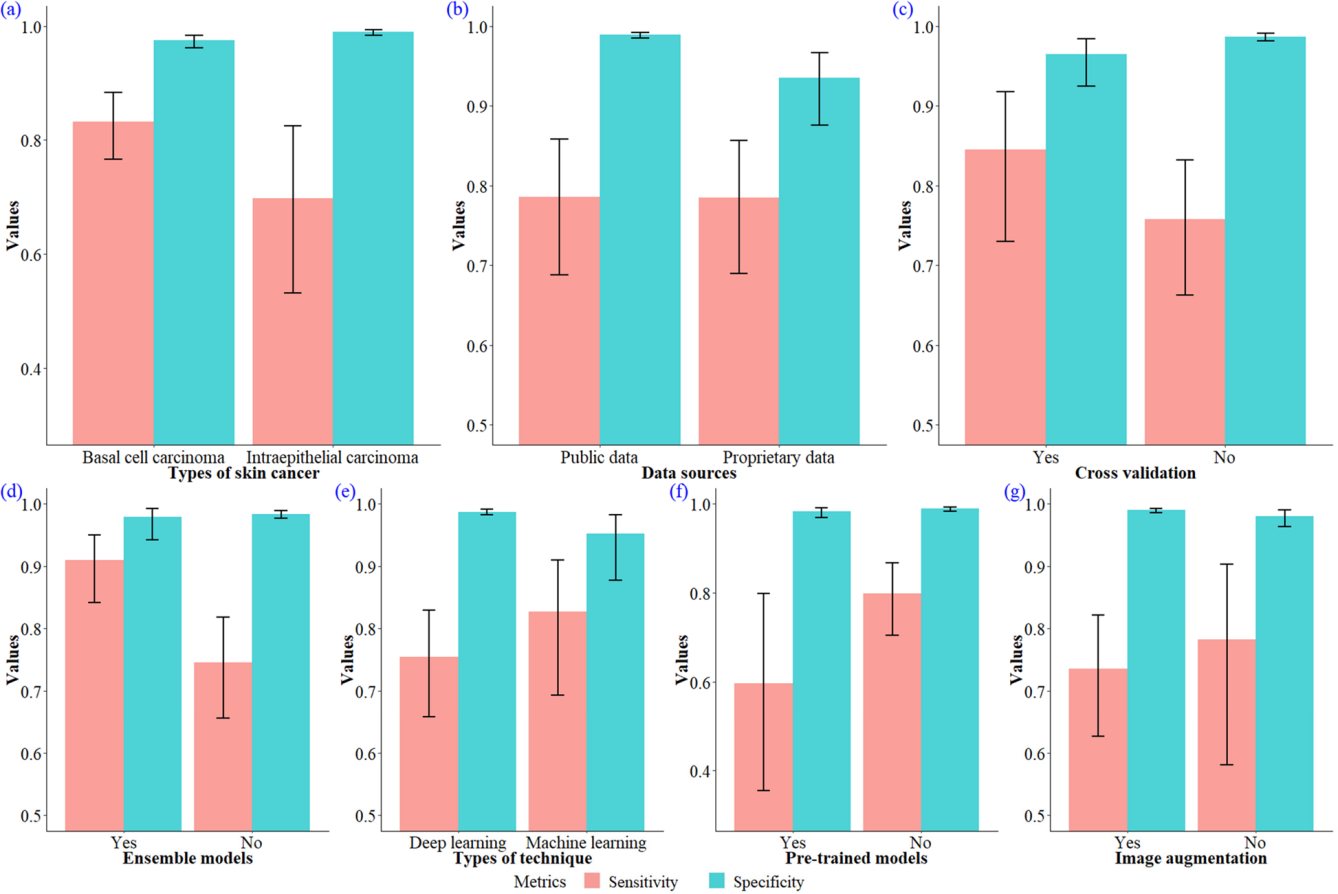
Summary sensitivity and specificity with 95% confidence interval for different covariates

As shown in Table 5 and Fig. 6(a), the sensitivity was higher for the studies classifying basal cell carcinoma (0.83; 95% CI, 0.77–0.88) than for the studies classifying intra-epithelial carcinoma (0.70; 95% CI, 0.53–0.82), and a statistically significance was evident (*p* = 0.046). The corresponding specificity was close between basal cell carcinoma and intra-epithelial carcinoma (0.98; 95% CI, 0.96–0.98 vs. 0.99; 95% CI, 0.98–0.99), but a significant difference was still detected (*p* = 0.006).

Studies utilized public data sources, as displayed in Table 5 and Fig. 6(b) achieved a higher sensitivity (0.79, 95% CI, 0.69–0.86 vs. 0.78, 95% CI, 0.69–0.86) than those used proprietary data sources, but didn’t reach statistical significance (*p* = 0.686). Specificity was however significantly higher for studies which adopted public data sources (0.99; 95% CI, 0.98–0.99) than proprietary data sources (0.93; 95% CI, 0.88–0.97, *p* < 0.001).

As presented in Table 5 and Fig. 6c), studies that implemented cross-validation procedures during the training phase had a higher sensitivity (0.79; 95% CI, 0.64–0.89 vs. 0.78; 95% CI, 0.69–0.85) than studies that did not, but no statistical significance was observed (*p* = 0.380). However, studies that implemented cross validation showed a significant lower specificity than studies that did not (0.97; 95% CI, 0.92–0.98 vs. 0.99; 95% CI, 0.98–0.99, *p* = 0.008).

Studies that adopted ensemble models, as depicted in Table 5 and Fig. 6(d), had a significant higher sensitivity (0.91; 95% CI, 0.84–0.95 vs. 0.75; 95% CI, 0.66–0.82, *p* = 0.014), and a tied specificity (0.98; 95% CI, 0.94–0.99 vs. 0.98; 95% CI, 0.98–0.99).

The studies that used deep-learning techniques, as showed in Table 5 and Fig. 6(e), achieved a lower sensitivity (0.75; 95% CI, 0.66–0.83 vs. 0.83; 95% CI, 0.69–0.91) and a higher specificity (0.99; 95% CI, 0.98–0.99 vs. 0.95; 95% CI, 0.88–0.98) than the studies that used machine-learning techniques. Significant differences were however only found in specificity (*p* = 0.001) but not sensitivity (*p* = 0.344).

As depicted in Table 5 and Fig. 6(f), deep learning studies that adopted pre-trained models achieved a higher sensitivity (0.81; 95% CI, 0.73–0.87 vs. 0.58; 95% CI, 0.33–0.79) and a higher specificity (0.99; 95% CI, 0.98–0.99 vs. 0.98; 95% CI, 0.97–0.99) than studies that did not. Statistical significant difference was only established for sensitivity (*p* = 0.048) rather than specificity (*p* = 0.263).

Table 5 and Fig. 6(g) showed that the sensitivity of the deep learning studies that adopted image-augmentation procedures to deal with over-fitting was lower than the studies without adopting image-augmentation procedures (0.73; 95% CI, 0.62–0.82 vs. 0.80; 95% CI, 0.61–0.91), but no statistical difference was observed (*p* = 0.413). There was however a statistically significant difference between studies adopted image-augmentation procedures and studies that did not (0.99; 95% CI, 0.99–0.99 vs. 0.98; 95% CI, 0.96–0.99, *p* = 0.031).

## Discussion

Considering the potential health threat posed by non-melanoma skin cancer and the advances of AI techniques made in recent years, it is timely to both synthesize and report the results in diagnosis of non-melanoma skin cancers based on AI. According to the included studies, our meta-analysis shows there to be diagnostic performance with a moderate sensitivity, a strong specificity and AUC. Sensitivity was significantly dependent on the types of non-melanoma skin cancer encountered and whether ensemble models were adopted, while specificity was significantly dependent on included covariates except for whether ensemble models were adopted and whether pre-trained models were used.

Prior studies [8, 9] show that the accuracy for diagnosing non-melanoma skin cancers based on AI is closer to human diagnosis based on sensitivity. It is also notable that the performance of AI is better than that based on human diagnosis in terms of specificity and AUC [8, 9]. However, the pooled sensitivity in this study is lower than the pooled sensitivity in diagnosing melanoma skin cancer based on AI. More studies are required to improve the performance of AI used to diagnose non-melanoma skin cancer, especially in terms of sensitivity. Significant covariates identified in this study may be included for purposes of this improvement.

In terms of differing types of non-melanoma skin cancer, the sensitivity of basal cell carcinoma was higher than those found in intra-epithelial carcinoma. Significant difference between basal cell carcinoma and intraepithelial carcinoma was observed. The potential reason that basal cell carcinoma reached a significant higher sensitivity may be due to more images of this type of skin cancer are included and thus more characteristics are learned than the other type of skin cancer (10,363 vs. 3,829). The specificity of basal cell carcinoma was lower than, but close to, that of intra-epithelial carcinoma. In words, there are few false positive results when using artificial intelligence to detect non-melanoma skin cancers.

Data for building machine-learning models that are collected from public data sources, in general, are more frequently leveraged than proprietary data sources in the present study. It is therefore reasonable that predictive models based on public data sources often perform better than those based on proprietary data sources since data from public sources are better understood than data from proprietary sources. Our study confirmed studies that data used from public sources performs better in terms of both sensitivity and specificity than those of data used from proprietary sources. This finding was also reported by Dick et al. [16], but their study focused on meta-analyzing the computer-aided diagnoses of melanoma skin cancers. The finding reported here suggests that the performance of predictive models using proprietary data sources may still have room for improvement by leveraging differing machine-learning or deep-learning techniques. Further, echoing the point of Tschandl et al. [3], more skin cancer images of all pertinent kinds should be collected and, most importantly, be made publicly available.

In order to prevent over-fitting [73], it is widely acknowledged to split collected data into a training dataset and a test one. The training dataset is used for training the predictive model while the test dataset is used to validate the established model [74]. To better estimate the model performance, cross- validation methods focused on the training dataset are usually adopted [73]. In this research, studies that adopted cross validation showed a non-significant, higher sensitivity but a significant, lower specificity than those that did not. One possible explanation for studies adopting cross-validation methods showed a significant, lower specificity that may contribute to different types of cross-validation methods. This explanation included threefold [44], fivefold [62, 68], tenfold [19, 24, 32, 51], or leave-one-out [13, 48, 59] that were adopted as part of these studies. As such, they may have caused a larger variation in specificity, as illustrated in Fig. 6c.

Ensemble models are predictive models that combine predictions from more than one model into one model [75]. They tend to have much better predictive performance than singular models [74]. It is therefore reasonable to assume predictive models based on ensemble models perform better than single models. Our study confirmed that studies based on ensemble models performed better in sensitivity (0.91 vs. 0.75) and a tied specificity (0.98 vs. 0.98) between two categories, a statistically significant difference was found only in sensitivity. This finding may indicate that ensemble models can be considered when utilizing AI for predicting non-melanoma skin cancers.

The summary sensitivity and specificity is (0.75, 0.99) and (0.83, 0.95) when deep-learning and machine-learning techniques were used, respectively; however, statistical significance difference was evident only in specificity. As illustrated in Table 4 and Fig. 6 (e), the 95% CIs of sensitivity of deep learning and machine learning are quite wide, indicating that where the real sensitivity lies is unsure, while the 95% CIs of specificity is quite narrow, suggesting the summary specificity is a more precise estimation. Regarding why the sensitivity of machine learning is higher than that of deep learning, we suspect the plausible reason may be due to most studies being based on a machine learning adopted support vector machine [26, 27, 29, 48, 59, 63] or random forest [19, 61]. The machine learning performances are generally considered as quite stable, while studies based on deep-learning techniques adopted a variety of differing techniques which may result in more unstable performance. Prior meta-analyses of diagnostic test accuracy of AI used for diagnosing melanoma skin cancers, by Dick et al. [16] and Rajpara et al. [23], showed summary sensitivities and specificities of (0.44, 0.92) and (0.91, 0.79), respectively. We however found that both deep-learning and machine-learning techniques performed moderately well with sensitivity and quite well with specificity. These inconsistent results are explainable by the fact that these meta-analyzed studies were not conducted in similar clinical settings. Further, both prior meta-analyses [16, 23] focused on melanoma rather than non-melanoma skin cancer types respective this study.

Pre-trained models are generalizable deep neural networks which are trained on large-scale data and can be transferred to many tasks [76]. Simply fine-tuning these pre-trained models with a small amount of task-related data can influence performance quite well [77]. Prior review evidence [36] also found pre-trained models outperformed experienced dermatologists. As such, deep-learning studies based on pre-trained models are expected to perform better than those that did not, as confirmed in this study. Both sensitivity and specificity of pre-trained models performed higher than those that did not, but only sensitivity reached statistical significance. This finding may imply that future studies may consider building predictive models based on pre-trained models to enhance predictive accuracy.

In order to prevent over-fitting, deep learning usually required large amounts of data for purposes of training [78]. Without sufficient training data, the generalization of predictive models may be limited [79]. Based on this notion, studies that adopted image-augmentation techniques are expected to perform better than those did not. Our study however showed that sensitivity is lower for studies that adopted image augmentation than those did not, while specificity is higher for studies adopted image augmentation than those that did not. Only specificity reached a statistically significant difference. One plausible reason for why studies adopted image augmentation achieved lower sensitivity than studies that did not is that the implemented-augmentation techniques were different among the included studies. Not every included studies utilized the same augmentation techniques, such as flipping, rotation, shearing, cropping, and translation.

Finally, our study meta-analyzed the diagnostic test accuracy based on PRISMA statement [38]. Common metrics for diagnostic test accuracy including area under receiver operating characteristic curve, sensitivity, specificity, diagnostic odds ratio, positive likelihood ratio and negative likelihood ratio were included. Furthermore, to account for the threshold effect, the pooled sensitivity and specificity was estimated based on a bivariate model [39]. Other metrics such as mean accuracy were not assessed in this study since prior evidence suggests that sensitivity and specificity are more sensible parameters to be analyzed in a meta-analysis, and they are clinically well known [80].

Just like most meta-analyses, our study has its limitations. First, the interpretation of summary sensitivity and specificity should be approached cautiously since heterogeneity among studies exists. Further, 72 studies were excluded due to insufficient quantitative information. Future diagnostic studies aimed at predicting non-melanoma skin cancers are suggested to include sufficient quantitative information for subsequent meta-analysis to better characterize and profile these studies. The covariates identified in this study are purely based from a statistical viewpoint [81], future research could consider the different design ideas of deep learning-based approaches or machine learning-based approaches to identify the incidence of other potential covariates. Finally, future meta-analysis may adopt emerging techniques [82–85] to cluster or classify models into different groups or categories, so that different insights are obtainable.

## Conclusions

Our study aims to meta-analyze the diagnostic test accuracy of applying AI techniques to the diagnosis of non-melanoma type skin cancer which is already considered insufficient in review evidence. Without a better understanding of the performance of AI for the diagnosis of non-melanoma skin cancer, the potential of AI may not be fully realized. Furthermore, the results of this quantitative meta-analysis can provide a more objective synthesis of the AI performance for diagnosing non-melanoma skin cancer. Based on the findings of this study, the usefulness of AI can be assessed with greater facility and objectivity. Moreover, strategies for improving the performance of AI used for screening non-melanoma skin cancer are identifiable. A quick, safe, and non-invasive screening of non-melanoma skin cancers can thus be expected. By searching multiple online databases, 39 articles (67 studies) were included for purposes of meta-analysis. A bivariate meta-analysis of diagnostic test accuracy was undertaken to obtain summary sensitivity, specificity, and AUC. A moderate diagnostic performance of summary sensitivity, a strong summary specificity, and a strong AUC were all observed based according to a bivariate meta-analysis of diagnostic accuracy test. Types of non-melanoma skin cancer, whether data sources were publicly available, whether cross-validation procedures were undertaken, whether ensemble models were adopted, the types of AI technique employed, whether pre-trained deep-learning models were used, and whether image-augmentation procedures were all determined to partially explain some of the heterogeneity found among primary studies. Future studies may consider adopting the suggested techniques to have better predictive performance of AI for the effective diagnosis of non-melanoma skin cancer.

## Supplementary Information

**Additional file 1.**

**Additional file 2.**

## Data Availability

The datasets used and analyzed during the current study are available from the corresponding author on reasonable request.

## Abbreviations

AI: Artificial intelligence
AUC: Area under receiver operating characteristic curve
BCC: Basal cell carcinoma
CI: Confidence interval
CNN: Convolutional neural network
DOR: Diagnostic odds ratio
DL: Deep learning
DTA: Diagnostic test accuracy
IEC: Intra-epithelial carcinoma
± LR: Positive/negative likelihood ratio
ML: Machine learning
ROC: Receiver operating characteristic curve
PRISMA: Preferred reporting items for a systematic review and meta-analysis statement
PI: Prediction interval
QUADAS-2: Revised quality assessment of diagnostic studies
