# Supplementary material for: The accuracy of artificial intelligence used for non-melanoma skin cancer diagnoses: a meta-analysis
Source: BMC Med Inform Decis Mak. 2023 Jul 28;23:138. doi: 10.1186/s12911-023-02229-w (PMC10375663; doi:10.1186/s12911-023-02229-w)
Supplement: Supplementary file 2 — Additional file 2. [file 12911_2023_2229_MOESM2_ESM.docx]

| **Section/topic** | **#** | **PRISMA-DTA for Abstracts Checklist item** | **Reported on page #** |
| --- | --- | --- | --- |
| **TITLE and PURPOSE** | | |  |
| Title | 1 | Identify the report as a systematic review (+/- meta-analysis) of diagnostic test accuracy (DTA) studies. | P. 1, Title |
| Objectives | 2 | Indicate the research question, including components such as participants, index test, and target conditions. | P. 1, Objective section |
| **METHODS** | | |  |
| Eligibility criteria | 3 | Include study characteristics used as criteria for eligibility. | P. 1, Methods section |
| Information sources | 4 | List the key databases searched and the search dates. | P. 1, Methods section |
| Risk of bias & applicability | 5 | Indicate the methods of assessing risk of bias and applicability. | P. 1, Methods section |
| Synthesis of results | A1 | Indicate the methods for the data synthesis. | P. 1, Methods section |
| **RESULTS** | | |  |
| Included studies | 6 | Indicate the number and type of included studies and the participants and relevant characteristics of the studies (including the reference standard). | P. 1, Results section |
| Synthesis of results | 7 | Include the results for the analysis of diagnostic accuracy, preferably indicating the number of studies and participants. Describe test accuracy including variability; if meta-analysis was done, include summary results and confidence intervals. | P. 1, Results section |
| **DISCUSSION** | | |  |
| Strengths and limitations | 9 | Provide a brief summary of the strengths and limitations of the evidence | N/A |
| Interpretation | 10 | Provide a general interpretation of the results and the important implications. | P. 1, Conclusions section |
| **OTHER** | | |  |
| Funding | 11 | Indicate the primary source of funding for the review. | See Funding section |
| Registration | 12 | Provide the registration number and the registry name | N/A |
